# A spatial analysis to study access to emergency obstetric transport services under the public private “Janani Express Yojana” program in two districts of Madhya Pradesh, India

**DOI:** 10.1186/1742-4755-11-57

**Published:** 2014-07-22

**Authors:** Yogesh Sabde, Ayesha De Costa, Vishal Diwan

**Affiliations:** 1 Department of Community Medicine, R.D. Gardi Medical College, Ujjain, Madhya Pradesh, India; 2 International Center for Health Research, R.D. Gardi Medical College, Ujjain, Madhya Pradesh, India; 3 Department of Public Health Sciences, Karolinska Institutet, Stockholm, Sweden; 4 Department of Public Health and Environment, R.D. Gardi Medical College, Ujjain, Madhya Pradesh, India

**Keywords:** Geographic information system, Spatial analysis, Access to health care, Equity in access, Emergency obstetric transport, India

## Abstract

**Background:**

The government in Madhya Pradesh (MP), India in 2006, launched “Janani Express Yojana” (JE), a decentralized, 24X7, free emergency transport service for all pregnant women under a public-private partnership. JE supports India’s large conditional cash transfer program, the “Janani Suraksha Yojana” (JSY) in the province and transports on average 60,000 parturients to hospital every month. The model is a relatively low cost one that potentially could be adopted in other parts of India and South Asia. This paper describes the uptake, time taken and geographic equity in access to the service to transport women to a facility in two districts of MP.

**Methods:**

This was a facility based cross sectional study. We interviewed parturients (n = 468) who delivered during a five day study period at facilities with >10 deliveries/month (n = 61) in two study districts. The women were asked details of transportation used to arrive at the facility, time taken and their residential addresses. These details were plotted onto a Geographic Information System (GIS) to estimate travelled distances and identify statistically significant clusters of mothers (hot spots) reporting delays >2 hours.

**Results:**

JE vehicles were well dispersed across the districts and used by 236 (50.03%) mothers of which 111(47.03%) took >2 hours to reach a facility. Inability of JE vehicle to reach a mother in time was the main reason for delays. There was no correlation between the duration of delay and distance travelled. Maps of the travel paths and travel duration of the women are presented. The study identified hot spots of mothers with delays >2 hours and explored the possible reasons for longer delays.

**Conclusions:**

The JE service was accessible in all parts of the districts. Relatively high utilization rates of JE indicate that it ably supported JSY program to draw more women for institutional deliveries. However, half of the JE users experienced long (>2 hour) delays. The delayed mothers clustered in difficult terrains of the districts. Additional support particularly for the identified hot spots, enhanced monitoring by state agencies and GIS tools can facilitate better effectiveness of the JE program.

## Background

### “Janani Suraksha Yojana” (JSY) or safe motherhood scheme

In order to improve population coverage of maternal health services, reduce inequity in access and move towards achievement of millennium development goal (MDG) 5 (reduction in maternal mortality), governments in South Asia have implemented innovative demand side financing initiatives over the last decade [1]. The most well-known of these is India’s large conditional cash transfer program, the “Janani Suraksha Yojana” (JSY) or Safe Motherhood Scheme [2,3], to reduce maternal mortality by promoting in-facility delivery. The Indian JSY is the largest conditional cash transfer in the world, with over 70 million beneficiaries since inception in 2005 [4]. The program, funded by the Government of India, pays women a fixed sum when they give birth in a facility. The cash transfer under the JSY attempts to reduce financial access barriers to accessing hospital delivery. The financial access barrier is closely linked to geographical access barriers to care, so that providing either funds for transport or providing transport itself reduces both financial and geographic barriers to access. Therefore, addressing the geographical access barrier and taking steps to address it, acts in synergy with the cash transfer like JSY to increase utilization of facilities for institutional birth.

### The importance of transport in the reduction of maternal mortality

Complications such as hemorrhage, hypertensive disorders, infections and unsafe abortions account for more than 50% of maternal deaths globally. Timely access to healthcare can prevent most of these fatal pregnancy complications [5-7]. The World Bank in its report (2008) suggests that 75% of maternal deaths could be prevented by timely access to emergency obstetric care (EmOC) [8]. Thaddeus and Maine has described three delays in access to obstetric care services viz. (1) delay the decision to seek care; (2) delay arrival at a health facility; and (3) delay the provision of adequate care. The delay in arriving at a facility after the decision has been made to seek care was referred to as the ‘second delay’ [9]. Lack of reliable transport is a major element contributing to this second delay [10]. A second delay of greater than 2 hours has been reported to be significantly associated with in-hospital maternal mortality [11-13]. It has been recommended that basic and comprehensive EmOC facilities should be available within two to three hours of travel for most women [14]. Given that the JSY runs in a low & middle income country (LMIC), and is strongly focused on poorer Indian provinces with high maternal mortality, the challenges to securing transport for delivery are many and include the absence of a reliable public transport system, particularly connecting the rural areas, poor road infrastructure, difficulty in arranging emergency transport at short notice, the lack of transportation alternatives to choose from and the extremely high cost of organizing such transport. Similar difficulties have been reported from other LMIC settings [15-17]. Therefore different innovative interventions to ensure an effective emergency transport have been investigated in these settings. In Mali and Sierra Leone, radios were provided to summon vehicles during obstetric emergencies [6,18]. A non-governmental organization, Transaid, works to improve management and maintenance of transport systems (including emergency obstetric transport systems) in LMICs in sub Sahran Africa, Sri Lanka and Afghanistan. They utilize professional transport operation experts to implement interventions such as professional training of the drivers, community managed transport and advocacy [19]. In Kenya, the transport costs were incorporated into the benefit package of a community insurance scheme [20]. In Malaysia and Sri Lanka, the health department engaged with the private sector to provide transport at subsidized rates for sending emergencies to hospitals [21]. In all these reports there was an improvement in the utilization rates and health outcomes (including obstetric outcomes) following provision of a better emergency transport system.

### “Janani Express Yojana” (JE) or maternal express program

To support access to institutional delivery under the JSY program, the department of health in the large central Indian province of Madhya Pradesh (MP) launched “Janani Express Yojana” (JE or maternal express program) in 2006 that covered the entire province by 2009. The JE was intended to make available emergency transport, so that mothers could travel to hospital for delivery. This innovative, decentralized, public-private partnership (PPP) was a 24×7 free emergency transport service available to all pregnant women in the province. Under the JE, the state, through its peripheral district health offices entered into agreements with local private transport operators to provide, when requisitioned emergency transport for pregnant women from their residence to obstetric care (OC) facilities for delivery. In this partnership, the service is paid for by the state, while private operators are responsible for vehicle procurment, maintenance and upkeep. A total of 893 vehicles operate under the JE in MP and the reports from the health department indicate that it has been used by 300,000 women to travel to the hospital to deliver since its inception. Currently, on average, the service transports 60,000 women in the province to hospital for delivery every month [22,23].

The JE service has been well utilized by the rural, tribal, and uneducated women. The high utilization of JE by all women including those from vulnerable groups suggests that the PPP has been able to provide a service where it is needed. However, available literature suggests that there is a need to study the effectiveness of the JE service from a geographic equity perspective i.e. whether women who reside in remote areas equally benefit from the scheme and to look at its efficiency in terms of time to transport JSY program beneficiaries [24]. Geographic information system (GIS) tools lend themselves to be used in such a study where distance and geographic location are key variables. The objectives of this paper are to study (i) the proportion of mothers delivering in facility who utilized the JE emergency transport service in two districts of MP, ii) the effectiveness of the JE service in terms of geographic equity in access using spatial analysis tools and iii) the efficiency of the service in terms of time i.e. time between the mother’s decision to go to hospital and reaching the hospital by the JE vehicle. The PPP model to provide transport to pregnant mothers to hospital for delivery is one that potentially could be adopted in other LMICs to improve access to EmOC. The results from this study are important both for the province that implements the program, other provinces in the Indian union and other similar settings looking for innovative alternatives to solve the problem of transportation for delivery.

## Methods

### Study area

**Figure 1:**
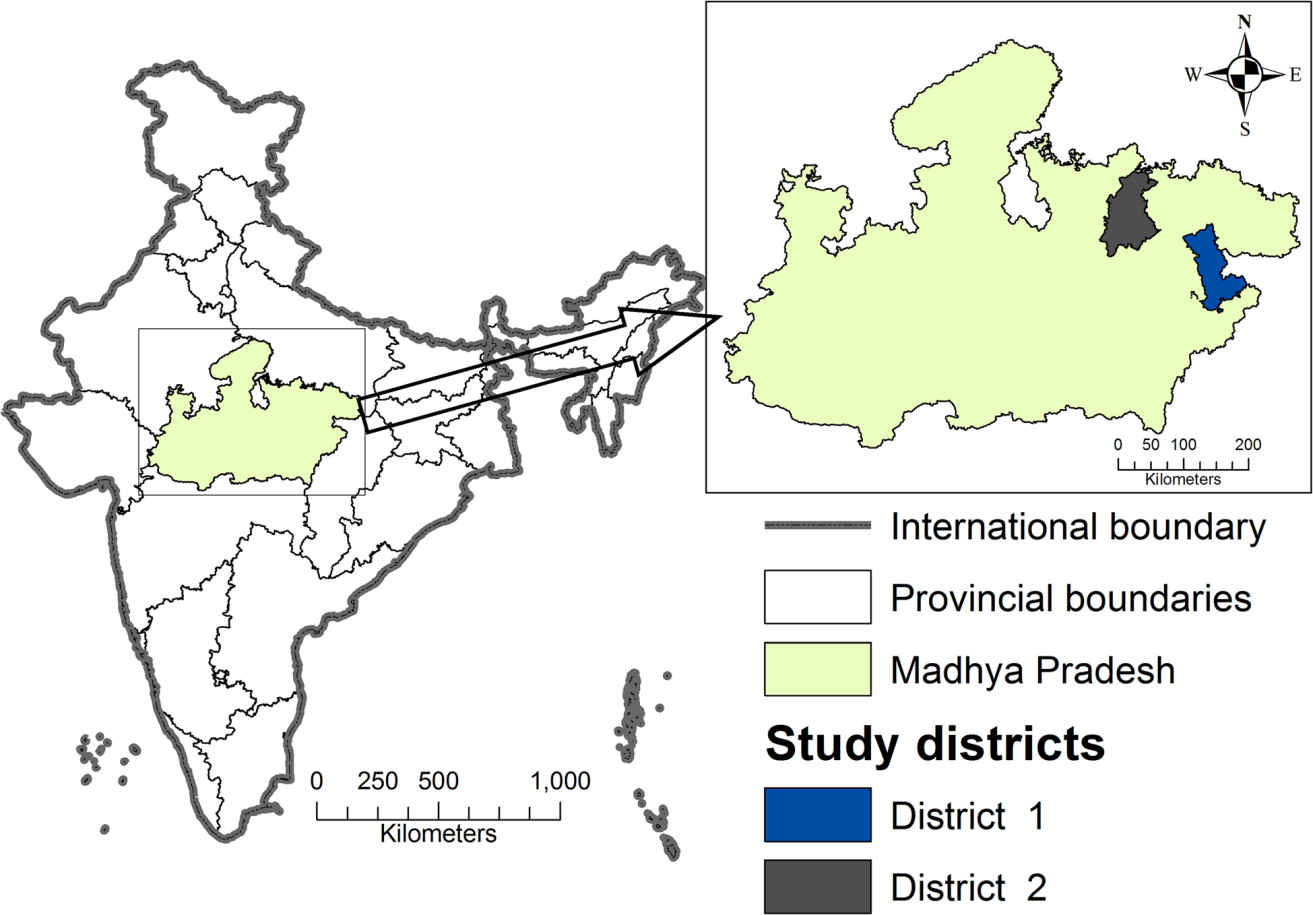
Map India showing location of province Madhya Pradesh and study districts.

The study was conducted in two districts of MP, India. MP is a large landlocked province (area 308,252 sq kilometers) (Figure 1) with a population of 72 million [25]. The infant mortality rate (IMR) (65 per 1,000 live births) and maternal mortality ratio (MMR) (277 per 100,000 live births) in MP are amongst the highest in the country [26]. MP is divided in to 51 administrative units called districts [27], each with population about 1 to 1.5 million. Each district has its own district health administration overseen by the provincial department of health. Districts are further subdivided into administrative blocks [25].

### The JE program

**Figure 2:**
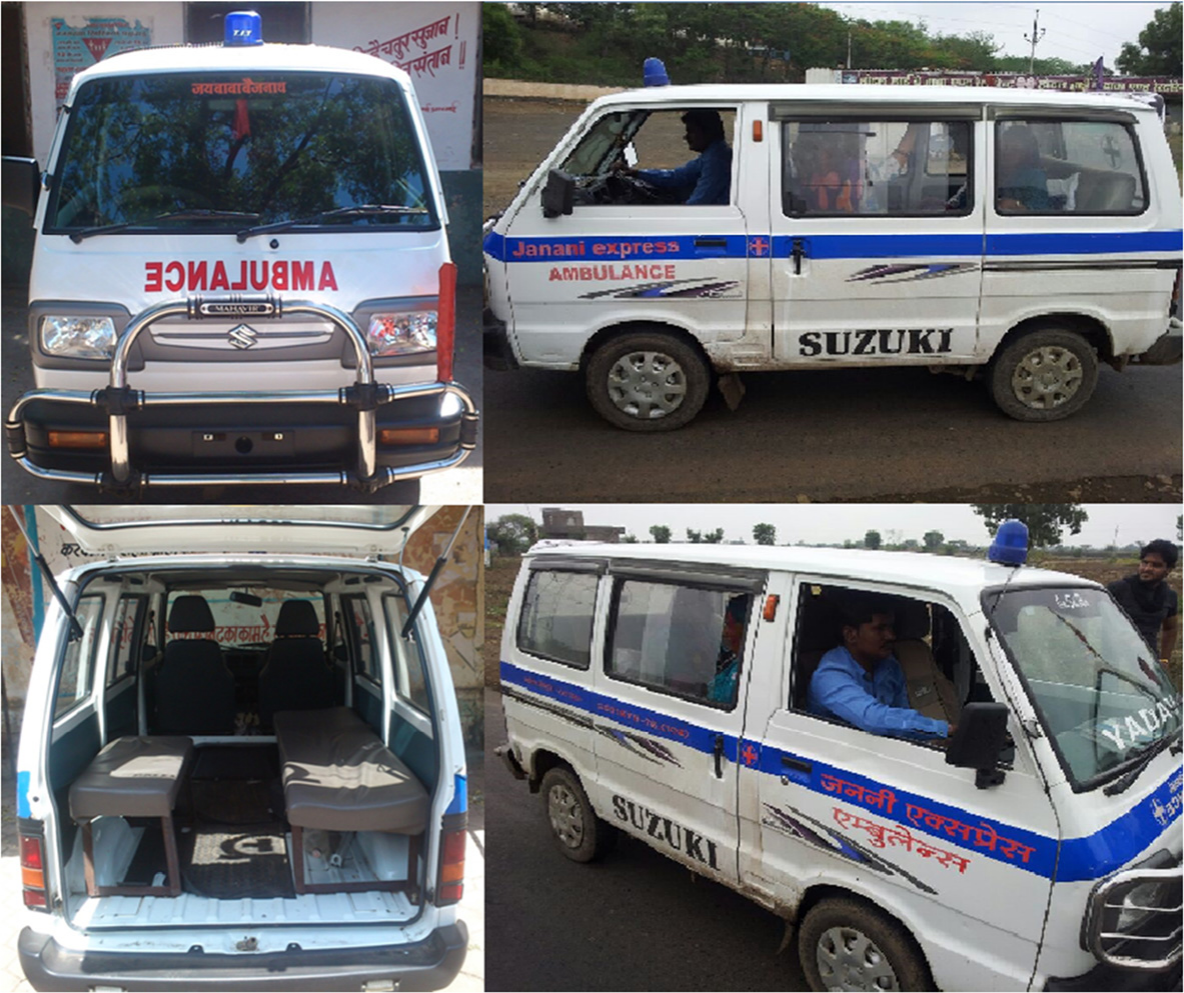
Photograph of Janani Express Yojana (JE) vehicle.

Under the JE program, each district health administration enters into contracts with private contractors located in the respective district, this contracting out is thus decentralized. The JE vehicles (vans that meet state stipulated specifications) (Figure 2) are owned and maintained by the private agencies that provide the service on behalf of the government. The JE vans are located across each district of the province, stationed at tertiary and some secondary public hospitals in the district. On average each administrative block within a district (0.1 million population approx.) has 2 to 3 vehicles under the scheme (i.e. 10–20 vehicles per district). Upon receipt of a telephone request made to a specially set up district call center located at the district head quarter, the nearest vehicle is dispatched to the woman’s location. Contractors are paid by the state based on the number of trips and distances traveled to bring the mothers to the hospital [22,23].

### Study districts

Two districts were purposively selected for this study. The districts each have a population 1.07 and 1.02 million respectively [25]. Both the districts have among the highest MMRs (435 and 397 per 100,000 live births respectively) within MP [28]; represent different population groups in the east and northeast part of the province (Figure 1). They have varying levels of socioeconomic development as mirrored by differences in their human development indices of 0.564 and 0.479 respectively [29]. In 2011, the respective proportion of institutional deliveries was 58% and 72% [28]. The public sector is the dominant provider of obstetric services.

### Study design

Facility based cross sectional study.

### Data collection

Data collection involved two parts (a) a facility survey and (b) interviews with parturients. For the facility survey, the list and addresses of public and private obstetric care facilities in each district was obtained from the district level health authorities. In addition, some facilities were identified during the actual field survey by snow balling. All identified facilities were visited by trained research assistants who, enquired about the number of deliveries conducted in the facility over the last three months. All facilities conducting more than 10 deliveries in a month were included in the study. The performance of Emergency Obstetric Care (EmOC) signal functions at the facilities was ascertained to allow their classification into Basic Emergency Obstetric Care (BEmOC) or Comprehensive Emergency Obstetric Care (CEmOC) facilities based on UNFPA classification [30]. ‘Non-CEmOC’ facilities were those that provided caesarean section services but failed to provide all eight CEmOC signal functions. ‘Non-BEmOCs’ were the facilities that failed to provide six basic EmOC signal functions. The JE call center in each district provided a list of the facilities where a JE vehicle was stationed in the respective districts.

For mothers’ interviews, a team of trained female research assistants visited all included facilities for five consecutive days between February 2012 and January 2013. All mothers delivered in the study facilities during the study period were interviewed to ask for their residential address and information on their journey to the facility for delivery. Mothers were requested to narrate the events leading up to delivery beginning from the noticing of labour pains through decisions to seek care and travel to the first OC facility. Interviewers probed to know the time of key events of interest including time when the decision was made to seek medical care, by whosoever was the decision maker in the particular family; time they started the journey to the facility and the time they reached the first facility. Mothers who took longer than 2 hours to reach the first OC facility after deciding to leave their home were identified as “long second delay” mothers and further interviewed to elicit reasons for their delay.

### Mapping

Geo-referenced data of the study districts was input in ArcMap version 10 (i) The boundary maps of the study districts and their villages (village maps) were obtained from the office of Survey of India. Geo-referencing of the boundary maps was done using Survey of India topological sheets of the scale 1:50,000. The geo-referencing was cross verified on the ground using hand held global positioning system (GPS) at random locations. (ii) The locations of obstetric care facilities and JE vehicle stations were digitized using the recordings obtained from hand held GPS. iii) The locations of the villages from where mothers travelled were identified from their residential addresses as provided to the research assistants in the survey. The locations of mothers’ villages were mapped onto the GIS.

### Analysis

A database was created in research electronic data capture (REDCap) [31]. Primary data was entered into REDCap and subsequently exported to STATA version 12 for data analysis. The duration of second delay (time required to arrive at a facility after the decision has been made to seek care) was calculated as the difference between the time of mothers’ decision to seek care and the time of actual arrival at the first OC facility. The duration of second delay was studied among JE users and non-users using median, inter quartile range (IQR) and compared using non parametric tests. The mothers with second delays longer than 2 hours were considered as “long second delay” mothers and their frequency was compared among JE users and non-users using percentage and odds ratio (95% CI).

### Geographic Information System (GIS) applications

ArcMap version 10 was used for GIS applications. Average Nearest Neighbor (Spatial Statistics) tool in ArcMap 10 was used to study the distribution of JE vehicles in the districts. The Average Nearest Neighbor tool measures the distance between each JE location and its nearest JE location. If the average distance between two neighboring JE locations was greater than a hypothetical random distribution, the JE locations were considered systematically dispersed. Network analysis was used to trace travelling routes of the mothers and distances travelled to reach facility. The travelled distances were correlated with the duration of travel and compared among subgroups of mothers using descriptive statistics and nonparametric tests. The Hot Spot Analysis (a spatial statistics tool) in ArcMap was used to calculate the Getis-Ord Gi* statistic for the duration of second delay for each mother taking >2 hours to reach facility. This tool works by looking at each feature within the context of neighboring features. In this the local sum for a mother and its neighbors is compared proportionally to the sum of all mothers and corresponding Z-score is calculated using Getis-Ord Gi* statistic for each mother (spot) on the map. The spots with statistically significant high values of positive Z-scores (above 1.96) are referred to as ‘hot spots’. To be a statistically significant hot spot, a mother will have a high value of delay and be surrounded by other mothers with high values of delay as well. When the local sum is very different from the expected local sum and that difference is too large to be the result of random chance, statistically significant z-score results. In this study ‘hot spots’ of mothers taking >2 hours to reach facility were located in both the districts to show the geographic areas where access to emergency transport was compromised [32].

### Ethical considerations

The study was approved by the institutional ethics committee of R.D.Gardi Medical College, Ujjain, MP, India. Written informed consent was taken from all study participants.

## Results

**Figure 3:**
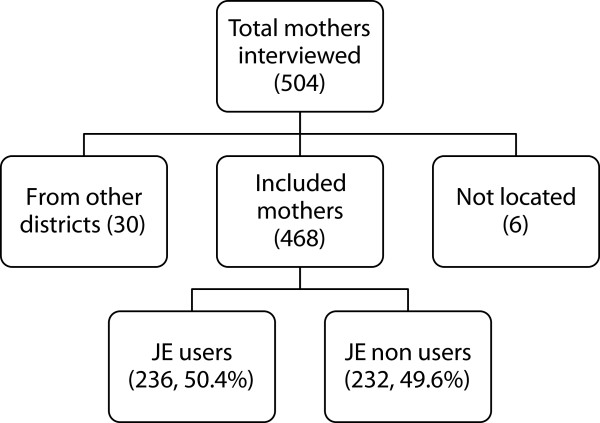
Flow chart showing study participants.

A total 62 obstetric care facilities performing > 10 deliveries/ month were identified in both the districts. All 62 facilities were visited of which 61 (55 public/ 6 private) consented to participate. Table 1 shows the ownership and EmOC status of the studied obstetric care facilities. Caesarean section (CEmOC and Non-CEmOC) services were provided by 8 of 61 facilities. District 1 had 7 Non-CEmOC facilities of which 6 were located in the district head quarter (HQ) town and one 20 kilometers (km) away from district HQ. District 2 had one CEmOC facility located at district HQ. There were no BEmOC facilities. The remaining 53 facilities were non-BEmOCs (27 and 26 in district 1 and 2 respectively). Most commonly missing signal functions were assisted vaginal delivery and manual removal of placenta. All the non-BEmOC facilities were in the public sector. There were a total of 32 JE vehicles in these two districts (13 in district 1 and 19 in district 2). They were stationed at 30 facilities.Figure 3 shows the participation of mothers in the study. A total of 504 mothers were interviewed from two districts of which, 30 mothers were excluded as their addresses were outside the district boundaries. The residences of 6 mothers could not be digitized as their addresses could not be located. Therefore a total of 468 (92.9%) mothers’ locations were digitized onto a GIS (221 from district 1 and 247 from District 2) of which 330 (70.51%) delivered in a non-BEmOC facility.

**Table 1:** Distribution of obstetric care facilities

**Facilities**	**Public (No.)**	**Private (No.)**
CEmOC	1	0
Non-CEmOC	1	6
Non-BEmOC	53	0
Total	55	6

### Distribution and uptake of OC facilities and JE vehicles

**Figure 4:**
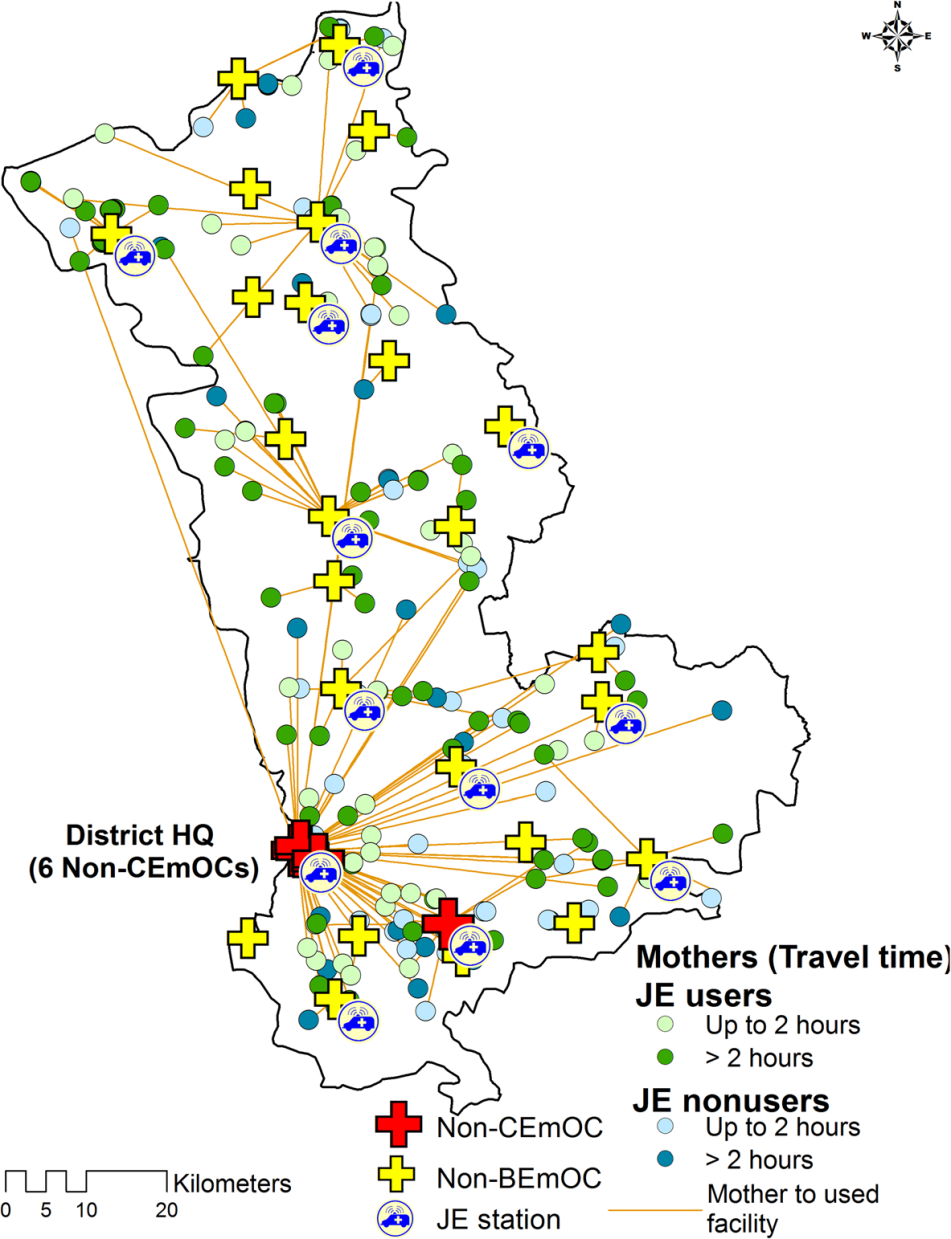
Distribution of institutional delivery mothers by their JE use and second delay in district 1.

**Figure 5:**
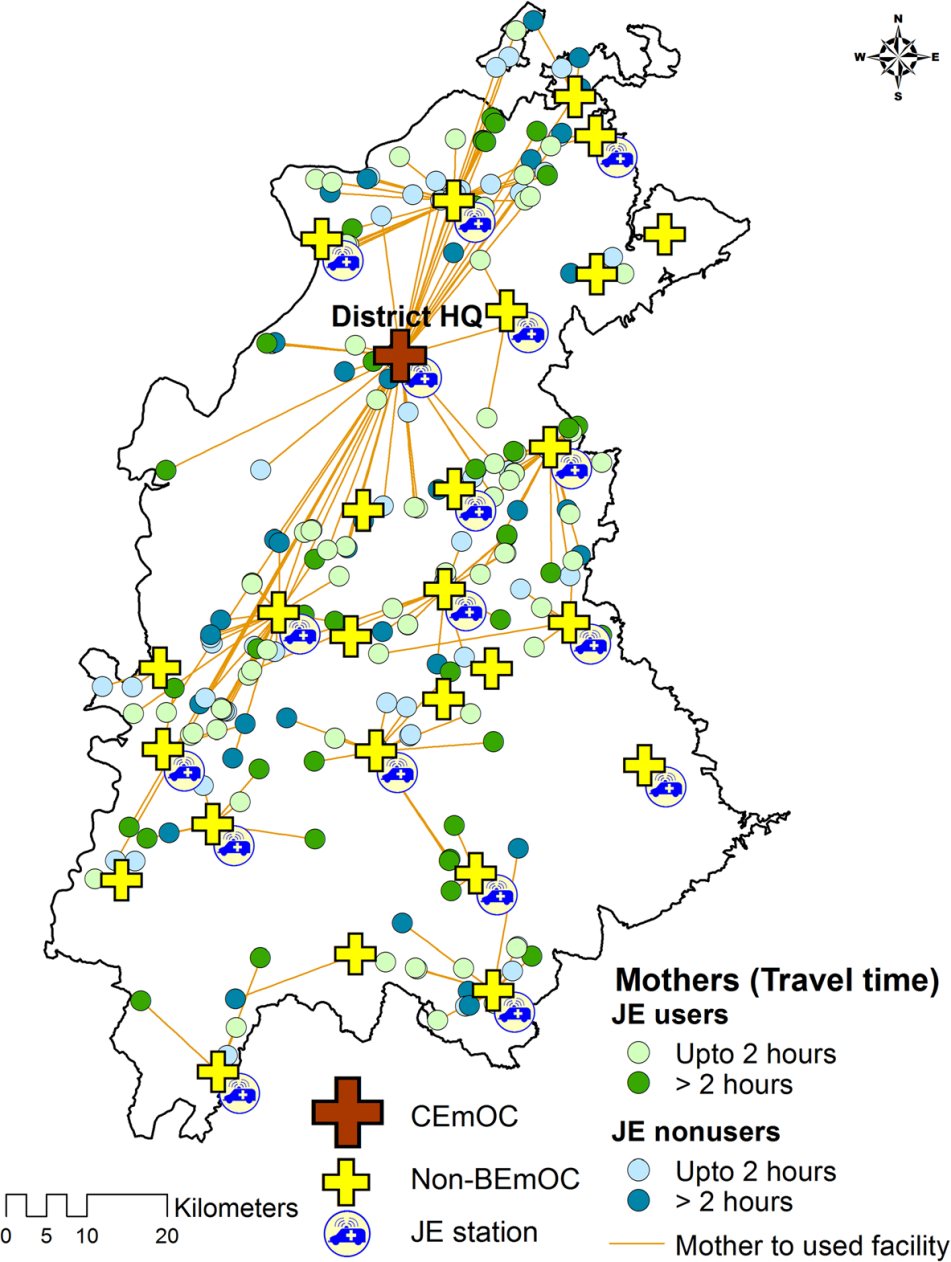
Distribution of institutional delivery mothers by their JE use and second delay in district 2.

Figures 4 and 5 illustrate the distribution of obstetric care facilities, JE vehicles and mothers in the two districts. The maps show that non-BEmOCs were distributed across all areas of the districts but the CEmOCs were located mainly at the district head quarters. In these maps (Figure 4 and 5) orange lines are used to link mothers with the facilities where they delivered. The orange lines show that some mothers bypassed non-BEmOCs on their way and preferred to deliver in district level facilities providing caesarean section services (CEmOCs and non-CEmOCs). The JE vehicles were used by 236 (50.43%) mothers. Only 2 (0.85%) JE users delivered in private facilities. Average Nearest Neighbor analysis revealed that of JE vehicles were systematically (not clustered or randomly) dispersed across the districts with nearest neighborhood ratios of 1.75 and 1.64 (p value < 0.001) respectively.

### The ‘second delay’

The median duration of second delay i.e. time needed to reach facility after making decision to seek care was 1.92 hours (1 hour 55 minutes) (IQR 1–4), similar in both districts. Only 55 (11.75%) mothers reached facility within one hour. A median second delay of 2 and 1.92 hours respectively was seen among both users and non-users of the JE vehicles.

### Magnitude of and reasons for long second delay

**Figure 6:**
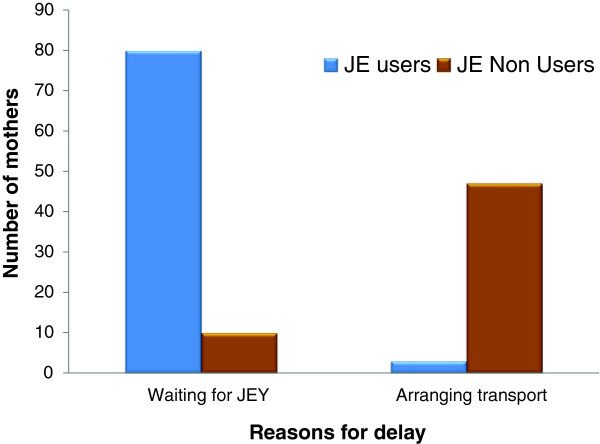
Bar diagram showing the frequency of different reasons for delay more than 2 hours.

Of the 468 mothers studied, 195 (41.7%) mothers took more than two hours to reach a facility after deciding to seek care. Majority of them (140, 71.79%) reported that poor availability of emergency transport services was responsible for their long second delay. The proportion of mothers with ‘long second delay’ was significantly higher among JE (111, 47.03%) users than JE non-users (84, 36.21%) (OR = 1.57, 95% CI = 1.08 - 2.27). The Figure 6 shows specific reasons for long second delay as reported by users and non-users of the JE vehicles. Among 111 JE users 80 (72.07%) mothers faced ‘long second delay’ due to the late arrival of JE vehicle. In addition among the 84 JE non-users, 10 (11.90%) reported that they could not get access to JE vehicle despite trying to use it. These 10 mothers called for the JE vehicle and waited for it to come. They finally arranged alternative transport and reached a facility. This created a ‘second delay’ of more than two hours in these cases. The proportion of women experiencing long second delays was significantly higher among those who delivered in non-BEmOC facilities (145, 44.07%) as compared to CEmOC facilities (50, 35.97%) (p < 0.001). 46 (9.83%) mothers were referred in by some other facility, of which 18 (39.13%) took > 2 hours to reach the first facility (long second delay mothers).

### Travelled distances (Network analysis)

The median distance travelled by the mothers to reach a facility was 9.53 kilometers (km) (IQR 4.38 – 19.10 km). The JE users travelled median 10.33 km (IQR 5.92 – 19.48). JE non-users travelled median 7.42 km (IQR 2.05 – 18.35). The JE users travelled significantly longer than JE non-users (two sample Wilcoxon rank-sum test p < 0.001). Table 2 shows the distances travelled according to the place of delivery among users and non users of JE. Only 23% of JE users delivered in a CEmOC/non-CEmOC facility compared to 37% of JE non-users (p value < 0.001). The mothers delivered in CEmOC/non-CEmOCs travelled significantly longer distances than those delivered in non-BEmOCs (Two sample Wilcoxon rank-sum test p < 0.001). There was no significant difference between the distances travelled by mothers with and without delivery complications. The mothers who were referred from one facility to another facility also did not travel significantly longer distances than unreferred mothers.

**Table 2:** Distribution of distance travelled by facility and JE use

	**CEmOC/Non-CEmOC**		**Non-BEmOC**	
**JE use**	**No. (%)**	**Distance travelled Median (IQR) km**	**No. (%)**	**Distance travelled Median (IQR) km**
JE User	54 (22.9*)	21.9** (14.1 – 34.4)	182 (77.1)	9.1 (5.5 – 16.1)
JE non user	85 (36.6*)	17.3** (1.8 – 30.2)	147 (63.4)	6.1 (2.5 – 12.8)

*χ^2^ test p value < 0.001.

**Wilcoxon rank-sum (Mann–Whitney) test p value < 0.001.

The duration of second delay showed very weak correlation with the length of travel (Spearman’s rho = 0.1509).

### Hot spot analysis

**Figure 7:**
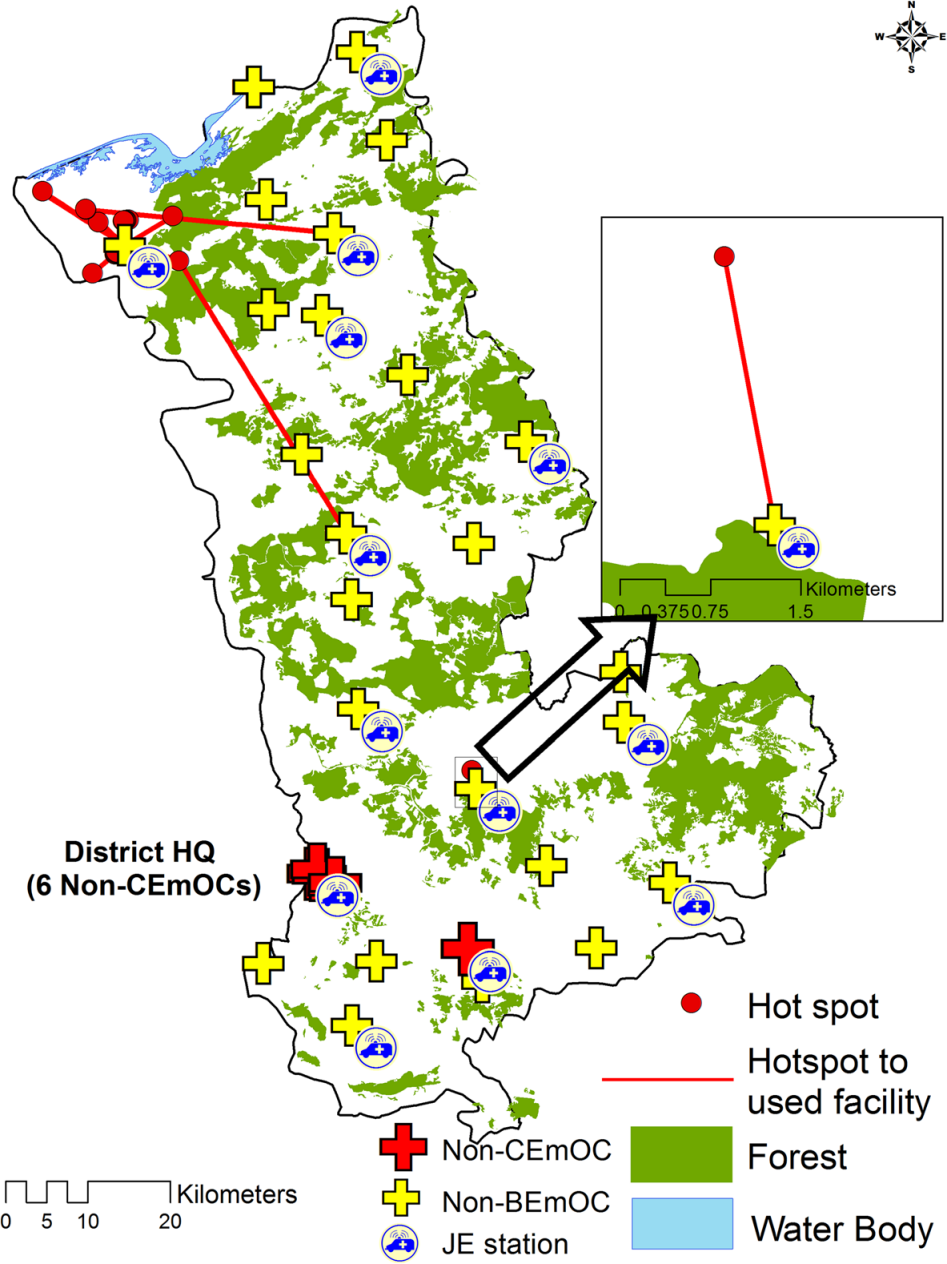
Distribution of hot spots in district 1 on the background of forest terrain.

**Figure 8:**
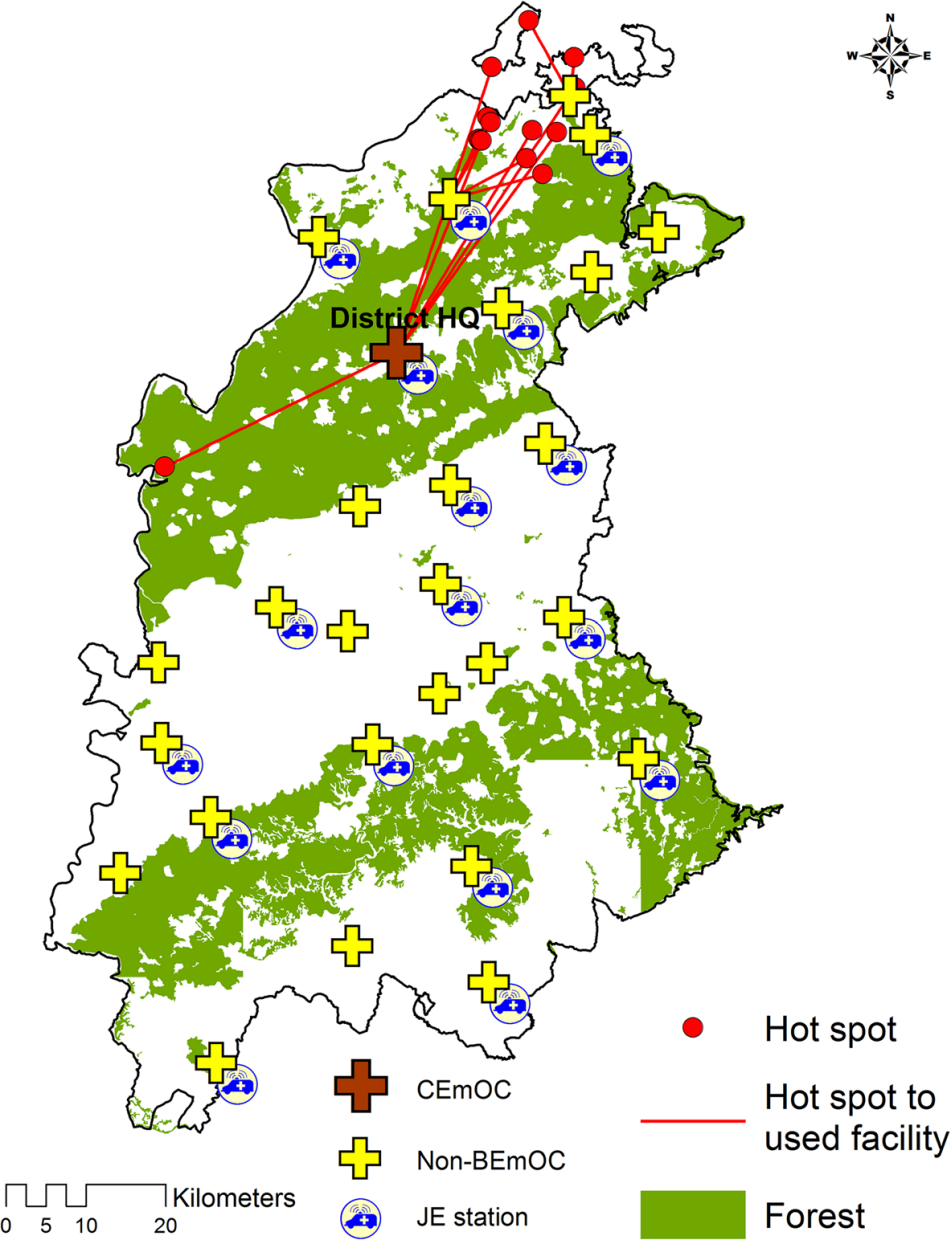
Distribution of hot spots in district 2 on the background of forest terrain.

The statistically significant hot spots of mothers experiencing long second delays (>2 hours) are shown in Figures 7 and 8 for the two study districts respectively. The median time taken by the mothers in hot spots was 6.50 and 5.25 hours in district 1 and 2 respectively. In district 1 most of the hot spot mothers delivered in a nearest facility which also had a JE station. The travelled distances of hot spot mothers (median = 5.50 km) were shorter than non-hot spot mothers (Median = 10.00 km). In district 2 most of the hot spot mothers delivered in CEmOC at district head quarter which was far away from them (median distance = 41.00 km). Most of the hot spots in district 2 were relatively close to the nearest non-BEmOCs (median distance = 10.60 km).

## Discussion

Emergency obstetric transport is an important barrier to accessing EmOC for many mothers in LMICs. JE is an important innovative intervention to ensure emergency transport access in LMICs, particularly because of the scale on which it has been implemented and has facilitated emergency obstetric transportation. The available reports on this large emergency transport program (JE) that critically supports the state’s conditional cash transfer program (JSY), suggest that there is a need to improve coverage and time efficiency of the JE service [24]. This paper explores the JE service for its utilization, geographic equity in access and effectiveness in transporting pregnant mothers to EmOC facilities. This study sheds light on these areas, particularly challenges and discusses areas for improvement which have implications for the organization of emergency transport systems for EmOC in other LMICs.

### What is the coverage of JE?

In our study the locations of JE vehicle stations were well dispersed in both the districts. Every second woman who delivered in a facility (50%) used a JE vehicle for her travel to the facility during labor. A study in 1999 reported that only 9 percent mothers in India used ambulances during labor [33]. Improvement in access to a functioning transport system is expected to improve utilization rates and subsequently clinical outcomes in the setting. For example, in Sierra Leone the provision of emergency transport services for women resulted in a higher proportion of project vehicle users arriving in better clinical condition than non-users [18]. Lack of geographic access, adversely affected the utilization of available EmOC services by rural women in Zambia [34]. Therefore in our setting, presence of JE vehicle as an accessible transport service is a key link to access and better utilization of maternal health services under JSY program. The relatively high proportion of users in this study shows that JE clearly fills a need.

### Does the JE reduce delay to accessing EmOC?

In our setting, 40% of mothers who delivered in the facilities could not reach a facility within two hours after making a decision to seek care (long second delay). Many mothers encountered long second delays in spite of using JE vehicles. In fact the JE users encountered long second delays more frequently than the JE non-users. This was mostly due to time taken by JE vehicle to reach the mothers’ residence. This shows that while JE non-users lose time trying to organize transport, the JE users lose time waiting for it. Ideally, JE should reduce the time before arrival of the transport because no organization or new arrangements to obtain a vehicle privately is required. But this potential gain is offset by long waiting times of JE. So from the mother’s point of view, though JE might save her money, in terms of time, she is still at risk of complications and death. An emergency transport vehicle that arrives late defeats the purpose of ‘emergency transport’ as delays can place the mother (and fetus) at significant risk of complication and death [33,35-37]. The present study was a hospital based study. It did not take into account those mothers who delivered at home or on their way to OC facility. Therefore the present study, cannot rule out the possibility that there might be a subset of mothers in the community who in spite of their willingness to deliver in a facility, delivered at home owing to non availability or late arrival of transport vehicle. These findings suggest that though the JE is a unique initiative and necessary complement to the JSY, it may be rendered less effective if it fails to transport pregnant mothers to hospital in time.

### Does long travelling distance influence long second delay?

Our analysis revealed that there was weak correlation between travelled distances and duration of second delay. The high proportion of “long second delay mothers” among JE users cannot be explained by distances that they travelled. It is more likely that the waiting time for the JE vehicle contributed to this delay.

### Does the access to EmOC facility and its level of functioning matter?

In this study, most facilities functioned at less than BEmOC levels. Most mothers (71%) delivered in such facilities. This was because non-BEmOCs were well dispersed in all areas of the districts and CEmOCs/non-CEmOC (fewer in number as compared to non-BEmOCs) were located only at the district headquarters. Mothers who delivered in CEmOC/non-CEmOC facilities travelled longer distances than those who delivered in the non-BEmOC facilities. The absence of key signal EmOC functions, indicate poor functioning of the peripheral non-BEmOC facilities; this is a possible explanation for this situation in which many mothers travelled significantly longer distances to deliver at CEmOC/non-CEmOCs located at district headquarters. Paradoxically, mothers who delivered at CEmOC/non-CEmOCs were less likely to experience long second delays. Ideally, CEmOC/non-CEmOC facilities are expected to be utilized by mothers with delivery complication who are referred by BEmOC facilities. But in our setting, there is no formal gatekeeping between different facility levels. Therefore a CEmOC/non-CEmOC facility can receive a large number of uncomplicated parturients who arrive directly from home (the majority), some parturients with complications who come directly from home or are referred from a lower level or private facility. Also there was no difference between the distances travelled by mothers with delivery complications and referred mothers. Given these observations, it is unlikely that mothers reached CEmOC/non-CEmOC facilities quicker because they were prioritized based on triage. It is possible that wealthier mothers who had the resources to organize non JE transport (hired or own) for themselves, bypassed dysfunctional lower level facilities (non-BEmOCs) and traveled directly to higher facilities (CEmOC/non-CEmOCs) at district head quarter taking lesser time, despite farther distances, than those delivered in non-BEmOCs. It is also likely that road networks are better leading upto CEmOC/non-CEmOC facilities (usually in the big towns) than lower level facilities which tend to be more remotely located.

In this study the 46 (9.83%) mothers were referred, of which 18(39.13%) took more than 2 hours to reach the first facility. Another study conducted in same setting reported that the average inter facility transfer time was 1.25 hours for referred cases [38]. The efficiency of emergency obstetric transport system is meaningful only if it can take pregnant mother to a facility where EmOC is available. In the given setting most of obstetric care facilities were not equipped with basic EmOC functions (non-BEmOCs). These facilities can possibly at the best handle completely uncomplicated deliveries but they need to refer any other deliveries to higher centers. In these cases the referral transport consumes vital time and adds to the delay in access to facility with EmOC services. Therefore unless peripheral facilities are made EmOC functional, transportation by itself has little meaning [6,39]. These findings indicate the need to strengthen non-BEmOC facilities in addition to improvement in referral and transport system in the given settings.

### Does the road condition contribute to the second delay?

Few decades ago the road density (road length per 100 square km of land area) in MP at 8.6 km/ 100 square km was lowest in India [40]. In 2000 a rural road development program was introduced in MP with the objective of building a strong infrastructure that would provide all-weather road access to every village/habitation with a population greater than 500. Since this initiative, overall road conditions have improved, however accessibility remains a problem for the more rural and remote villages [41]. The actual contribution of road conditions in the second delay was not explored in this study.

### What were the factors influencing hot spots?

Though women with long second delays were reported from all areas of both the districts, identified hot spots indicate that they clustered significantly in certain geographic areas. In the given setting there were many possible explanations for the long second delay among JE users as well as non-users. Available studies in similar settings report that the delay can be a result of long travelling distances, logistic factors like poor operational management system (communication, compliance by concerned personnel, availability and readiness of vehicles), inadequate vehicles and poor road infrastructure, that were investigated further for hot spots in each district [9,22,42,43]. In district 1, forest area covered 33.7% of the total district area (Figure 7). The geographic terrain of the hot spot was surrounded by forest on southern side and water bodies on northern side. The mothers rather than crossing this difficult terrain tended to deliver in the nearest facility which was a non-BEmOC equipped with JE vehicle. In this district, the mothers encountered long second delays in spite of using the nearest facility. The reason for this is possibly the difficult terrain, which the conventional two wheel drive van type vehicles operating under the JE find difficult to navigate despite short distances. These remote mothers can possibly benefit by having vehicles more able to travel across difficult terrain, in facilities close to such areas. The JE program also has a provision to establish linkages for the difficult areas [44]. In district 2, forests covered 52.4% of the total district area (Figure 8). Most of the hot spot mothers in district 2 acted differently in that they travelled longer distances through the forest areas to access the CEmOC located in the district head quarter. The majority of women will not require to deliver in a Comprehensive EmOC facility, but the alternative to not delivering in a CEmOC facility in this setting is nearly equivalent to delivering in a dysfunctional facility, as none of the other facilities provide complete Basic EmOC which is life saving. Therefore promotion of BEmOC deliveries must be preceded by improving the quality of care at the peripheral facilities not capable of providing BEmOC services at the time of study.

### Why did mothers bypass non-BEmOC facilities?

Figures 4 and 5 revealed that some mothers bypassed non functional BEmOCs (Non-BEmOCs) to seek care in higher (district level) facilities offering caesarean section services (CEmOCs and Non-CEmOCs). Bypassing primary care facilities to seek care in higher level facilities has been reported from other developing countries. This bypassing is costly and inefficient for pregnant mothers and the health system as it delays access to EmOC services, particularly for those residing in distant locations. Perceived poor quality of care is an important determinant for bypassing behavior [45-47].

### How can GIS tools support improvement in emergency obstetric transportation services?

The appropriate placement of adequate numbers of vehicles and monitoring of these vehicles is crucial to maximize the overall efficiency of the service, to improve accessibility for all pregnant women and minimize the second delay [42,43]. The JE program also has a provision to install GPS (global positioning systems) in the program vehicles, which can be harnessed for better monitoring of the vehicles [44]. However, interventions to improve emergency transportation demand rational use of resources particularly in LMIC settings like India. GIS has been effectively used to identify appropriate locations for new health centers, and emergency care services [48-51]. Studies also document the use of GIS in exploring and improving the access to obstetric care services [34,52,53]. But this is the first study in which GIS tools were used to identify potential areas for further interventions to increase the effectiveness of the emergency obstetric transport system.

### Methodological considerations

This study only includes women who ultimately gained access to transport as they were recruited after delivering in health facility. Additional ‘hot spots’ may exist for women who could not avail transport to a facility for delivery. To address this issue we plan to undertake further research at community level to explore the transport issues with mothers who delivered at home. We recorded the second delay as time from the when the decision to go to a facility for delivery to the time of arrival at the first facility. This time variable could not be split into time spent waiting for the vehicle to arrive and the actual time spent travelling on the road. All times were self reported by the mothers interviewed in the hospital, and therefore may have some biases related to the mothers perception of the time. The contribution of various non JE modes of transport and actual road conditions in the second delay was not explored in this study.

## Conclusions

The study reported relatively high utilization rates of JE, indicating that the JE program ably supported the JSY program in the province to draw more women into institutions for delivery. The JE service was accessible in all parts of the districts but users faced delays in reaching facilities. Failure of JE vehicles to reach in time at the mothers’ residence was one of the major contributors in this barrier, as there was no relationship between the distance from facility and the duration of the second delay. The poor performance of EmOC functions at the peripheral facilities was an important concern in our setting. There was significant clustering of the delayed mothers in certain areas (hot spots) of the districts. The findings of the study can assist in implementing targeted emergency transport interventions for better impact. Detailed regular review and assessment of JE in view of access, contact, promptness in response and patient satisfaction is a must. The state needs to play a strong role in monitoring the performance of the PPP – not just in terms of the number of women utilizing the service, but also in terms of quality of the service in terms of time efficiency. This may be supported with information technology and GIS. The identified hot spots should be given support by using additional resources and activities incorporated into JE to make it more efficient.

## Competing interests

The authors declare that they have no competing interests.

## Authors’ contributions

All the authors YS, ADC and VD contributed equally in conceptualization and planning of the study. YS and VD planned and executed data collection. YS managed the data entry, GIS mapping and GIS analysis. All authors reviewed and gave critical comments on the manuscript. All authors read and approved the final manuscript.
